# Supplementary material for: The role of lifestyle in mitigating cognitive decline in older adults with cardiometabolic multimorbidity (CMM): A protocol of systematic review and meta-analysis
Source: PLoS One. 2025 Dec 18;20(12):e0338896. doi: 10.1371/journal.pone.0338896 (PMC12714272; doi:10.1371/journal.pone.0338896)
Supplement: S2 File — (DOCX) [file pone.0338896.s002.docx]

## **S2 file: Search strategy**

### **EMBASE**

| **Main Concepts** | **Search** | **Query** | **Results retrieved** |
| --- | --- | --- | --- |
| **Cardiometabolic Multimorbidity (CMM)** | **#1** | **Index terms:**  ('cardiometabolic risk factor'/de OR 'cardiovascular disease'/de OR 'multiple chronic conditions'/de OR 'comorbidity'/de) AND ('diabetes mellitus'/mj OR 'cerebrovascular accident'/exp/mj OR 'hypertension'/mj OR 'hyperlipidemia'/mj OR 'dyslipidemia'/mj OR 'heart disease'/exp/mj) | 119,806 |
|  | **#2** | **Keywords (title or abstract):**  (cardiometabolic*:ab,ti OR metabolic*:ab,ti OR cardiovascular*:ab,ti OR multimorbid*:ab,ti OR comorbid*:ab,ti OR 'multiple chronic conditions':ab,ti) AND (stroke*:ab,ti OR 'heart disease*':ab,ti OR 'angina pectoris':ab,ti OR 'heart arrhythmia*':ab,ti OR ‘cardiac arrythmia*’:ab,ti OR 'atrial fibrillation':ab,ti OR 'heart failure':ab,ti OR 'heart infarction':ab,ti OR hyperlipid*:ab,ti OR dyslipid*:ab,ti OR diabet*:ab,ti OR hypertensi*:ab,ti) | 710,153 |
|  | **#3** | **#1 OR #2** | 759,422 |
| **Lifestyles** | **#4** | **Index terms:**  'lifestyle'/exp/mj OR 'health behavior'/exp/mj OR 'nutrition'/exp/mj OR 'sleep'/mj OR 'physical activity'/mj OR 'sport'/exp/mj OR 'exercise'/exp/mj OR 'smoking'/mj OR 'drinking behavior'/mj | 1,895,559 |
|  | **#5** | **Keywords (title or abstract):**  lifestyle*:ab,ti OR exercis*:ab,ti OR 'physical* activ*':ab,ti OR 'physical* inactiv*':ab,ti OR sport*:ab,ti OR sedentary:ab,ti OR diet*:ab,ti OR nutrition*:ab,ti OR 'alcohol use':ab,ti OR 'tobacco use':ab,ti OR sleep*:ab,ti OR leisure*:ab,ti OR recreation*:ab,ti OR 'social connect*':ab,ti OR 'social engag*':ab,ti OR 'social network*':ab,ti OR 'social activit*':ab,ti OR (((health OR eating OR drinking OR smoking) NEAR/2 (behav* OR habit*)):ab,ti) | 2,765,561 |
|  | **#6** | **#4 OR #5** | 3,763,098 |
| **Cognitive decline in longitudinal observational studies** | **#7** | **Index terms:**  ('alzheimer disease'/exp OR 'dementia'/exp OR 'cognitive defect'/exp OR 'cognitive reserve'/de OR 'cognitive resilience'/de OR 'cognitive aging'/de)  AND ('longitudinal study'/exp OR 'prospective study'/exp OR 'cohort analysis'/exp OR 'retrospective study'/exp OR 'case control study'/exp) | 82,769 |
|  | **#8** | **Keywords (title or abstract):**  (dementia*:ab,ti OR alzheimer*:ab,ti OR 'mild cognitive impairment' OR 'lewy body disease':ab,ti OR 'brain age':ab,ti OR 'memory loss':ab,ti OR 'memory disorder':ab,ti OR ‘neurocognitive disorder*’:ab,ti OR neurodegenerat*:ab,ti OR (cogniti* NEAR/3 (declin* OR deteriorat* OR defect* OR disorder* OR impair* OR dysfunction* OR perform* OR function* OR health OR aging OR reserve OR resilien*)):ab,ti) AND ('longitudinal*':ab,ti OR 'predict*':ab,ti OR 'prospective*':ab,ti OR 'follow up':ab,ti OR 'retrospective*':ab,ti OR 'wave':ab,ti OR 'time*point':ab,ti OR 'traject*':ab,ti OR 'over*time':ab,ti OR 'cohort*':ab,ti OR 'incidence':ab,ti OR 'hazard ratio':ab,ti) | 225,834 |
|  | **#9** | **#7 OR #8** | 251,821 |
| **Combined** | **#10** | **#3 AND #6 AND #9** | 2,884 |

### **Medline**

| **Main Concepts** | **Search** | **Query** | **Results retrieved** |
| --- | --- | --- | --- |
| **Cardiometabolic Multimorbidity (CMM)** | **#1** | **Index terms:**  ((MH "Cardiovascular Diseases") OR (MH "Cardiometabolic Risk Factors") OR (MH "Multimorbidity") OR (MH "Multiple Chronic Conditions") OR (MH "Comorbidity+")) AND ((MM "Stroke") OR (MM "Hyperlipidemias") OR (MM "Dyslipidemias") OR (MM "Diabetes Mellitus") OR (MM "Diabetes Mellitus") OR (MM "Hypertension") OR (MM "Heart Diseases+")) | 50,782 |
|  | **#2** | **Keywords (title or abstract):**  TI ( (cardiometabolic* OR cardiovascular* OR metabolic* OR multimorbid* OR comorbid* OR "multiple chronic conditions") AND (stroke* OR “heart disease*” OR “angina pectoris” OR “heart arrhythmia*” OR “cardiac arrhythmia*” OR “atrial fibrillation” OR “heart failure” OR “heart infarction” OR hyperlipid* OR dyslipid* OR diabet* OR hypertensi*) ) OR AB ( (cardiometabolic* OR cardiovascular* OR metabolic* OR multimorbid* OR comorbid* OR "multiple chronic conditions") AND (stroke* OR “heart disease*” OR “angina pectoris” OR “heart arrhythmia*” OR “cardiac arrhythmia*” OR “atrial fibrillation” OR “heart failure” OR “heart infarction” OR hyperlipid* OR dyslipid* OR diabet* OR hypertensi*) ) | 412,061 |
|  | **#3** | **#1 OR #2** | 437,578 |
| **Lifestyles** | **#4** | **Index terms:**  (MM "Life Style+") OR (MM "Health Behavior+") OR (MM "Diet+") OR (MM "Sleep") OR (MM "Exercise+") OR (MM "Sports+") OR (MM "Smoking") OR (MM "Alcohol Drinking") | 810,556 |
|  | **#5** | **Keywords (title or abstract):**  TI ( lifestyle* OR exercis* OR "physical* activ*" OR "physical* inactiv*" OR sport* OR sedentary OR diet* OR nutrition* OR "alcohol use" OR "tobacco use" OR sleep* OR leisure* OR recreation* OR “social connect*” OR “social engag*” OR "social network*" OR "social activit*" OR ((health OR eating OR drinking OR smoking) N2 (behav* OR habit*)) ) OR AB ( lifestyle* OR exercis* OR "physical* activ*" OR "physical* inactiv*" OR sport* OR sedentary OR diet* OR nutrition* OR "alcohol use" OR "tobacco use" OR sleep* OR leisure* OR recreation* OR “social connect*” OR “social engag*” OR "social network*" OR "social activit*" OR ((health OR eating OR drinking OR smoking) N2 (behav* OR habit*)) ) | 2,081,819 |
|  | **#6** | **#4 OR #5** | 2,473,984 |
| **Cognitive decline in longitudinal observational studies** | **#7** | **Index terms:**  ((MH "Cognitive Dysfunction") OR (MH "Cognition Disorders") OR (MH "Dementia+") OR (MH "Alzheimer Disease") OR (MH "Frontotemporal Dementia") OR (MH "Neurodegenerative Diseases") OR (MH "Neurocognitive Disorders") OR (MH "Cognitive Aging") OR (MH "Memory Disorders") OR (MH "Cognitive Reserve")) AND ((MH "Longitudinal Studies") OR (MH "Case-Control Studies+") OR (MH "Cohort Studies+") OR (MH "Prospective Studies") OR (MH "Retrospective Studies") OR (MH "Follow-Up Studies")) | 51,251 |
|  | **#8** | **Keywords (title or abstract):**  TI ( (dementia* OR alzheimer* OR “mild cognitive impairment*” OR “lewy body disease” OR “brain age” OR “memory loss” OR “memory disorder*” OR “neurocognitive disorder*” OR neurodegenerat* OR (cogniti* N3 (declin* OR defect* OR disorder* OR impair* OR dysfunction* OR perform* OR function* OR health OR aging OR reserve OR resilien*))) AND (longitudinal* OR predict* OR prospective* OR “follow up” OR retrospective* OR wave OR time*point OR traject* OR “over*time” OR cohort* OR incidence OR “hazard ratio”) ) OR AB ( (dementia* OR alzheimer* OR “mild cognitive impairment*” OR “lewy body disease” OR “brain age” OR “memory loss” OR “memory disorder*” OR “neurocognitive disorder*” OR neurodegenerat* OR (cogniti* N3 (declin* OR defect* OR disorder* OR impair* OR dysfunction* OR perform* OR function* OR health OR aging OR reserve OR resilien*))) AND (longitudinal* OR predict* OR prospective* OR “follow up” OR retrospective* OR wave OR time*point OR traject* OR “over*time” OR cohort* OR incidence OR “hazard ratio”) ) | 147,825 |
|  | **#9** | **#7 OR #8** | 168,566 |
| **Combined** | **#10** | **#3 AND #6 AND #9** | 1,466 |

### **CINAHL**

| **Main Concepts** | **Search** | **Query** | **Results retrieved** |
| --- | --- | --- | --- |
| **Cardiometabolic Multimorbidity (CMM)** | **#1** | **Index terms:**  ((MH "Cardiovascular Diseases") OR (MH "Cardiovascular Risk Factors+") OR (MH "Cardiometabolic Risk Factors") OR (MH "Comorbidity")) AND ((MM "Stroke") OR (MM "Hyperlipidemias") OR (MM "Dyslipidemias") OR (MM "Diabetes Mellitus") OR (MM "Diabetes Mellitus") OR (MM "Hypertension") OR (MM "Heart Diseases+")) | 25,178 |
|  | **#2** | **Keywords (title or abstract):**  TI ( (cardiometabolic* OR cardiovascular* OR metabolic* OR multimorbid* OR comorbid* OR "multiple chronic conditions") AND (stroke* OR “heart disease*” OR “angina pectoris” OR “heart arrhythmia*” OR “cardiac arrhythmia*” OR “atrial fibrillation” OR “heart failure” OR “heart infarction” OR hyperlipid* OR dyslipid* OR diabet* OR hypertensi*) ) OR AB ( (cardiometabolic* OR cardiovascular* OR metabolic* OR multimorbid* OR comorbid* OR "multiple chronic conditions") AND (stroke* OR “heart disease*” OR “angina pectoris” OR “heart arrhythmia*” OR “cardiac arrhythmia*” OR “atrial fibrillation” OR “heart failure” OR “heart infarction” OR hyperlipid* OR dyslipid* OR diabet* OR hypertensi*) ) | 101,718 |
|  | **#3** | **#1 OR #2** | 117,224 |
| **Lifestyles** | **#4** | **Index terms:**  (MM "Life Style+") OR (MM "Health Behavior+") OR (MM "Diet+") OR (MM "Sleep") OR (MM "Exercise+") OR (MM "Sports+") OR (MM "Alcohol Drinking") OR (MM "Smoking") OR (MM "Food Habits") | 449,580 |
|  | **#5** | **Keywords (title or abstract):**  TI (lifestyle* OR exercis* OR "physical* activ*" OR "physical* inactiv*" OR sport* OR sedentary OR diet* OR nutrition* OR "alcohol use" OR "tobacco use" OR sleep* OR leisure* OR recreation* OR “social connect*” OR “social engag*” OR "social network*" OR "social activit*" OR ((health OR eating OR drinking OR smoking) N2 (behav* OR habit*)) ) OR AB ( lifestyle* OR exercis* OR "physical* activ*" OR "physical* inactiv*" OR sport* OR sedentary OR diet* OR nutrition* OR "alcohol use" OR "tobacco use" OR sleep* OR leisure* OR recreation* OR “social connect*” OR “social engag*” OR "social network*" OR "social activit*" OR ((health OR eating OR drinking OR smoking) N2 (behav* OR habit*))) | 716,493 |
|  | **#6** | **#4 OR #5** | 964,290 |
| **Cognitive decline in longitudinal observational studies** | **#7** | **Index terms:**  ((MH "Dementia+") OR (MH "Cognition Disorders") OR (MH "Neurodegenerative Diseases") OR (MH "Neurocognitive Disorders") OR (MH "Cognitive Aging") OR (MH "Memory Disorders")) AND ((MH "Prospective Studies+" OR (MH "Retrospective Design")) | 16,585 |
|  | **#8** | **Keywords (title or abstract):**  TI ( (dementia* OR alzheimer* OR “mild cognitive impairment*” OR “lewy body disease” OR “brain age” OR “memory loss” OR “memory disorder*” OR “neurocognitive disorder*” OR neurodegenerat* OR (cogniti* N3 (declin* OR defect* OR disorder* OR impair* OR dysfunction* OR perform* OR function* OR health OR aging OR reserve OR resilien*))) AND (longitudinal* OR predict* OR prospective* OR “follow up” OR retrospective* OR wave OR time*point OR traject* OR “over*time” OR cohort* OR incidence OR “hazard ratio”) ) OR AB ( (dementia* OR alzheimer* OR “mild cognitive impairment*” OR “lewy body disease” OR “brain age” OR “memory loss” OR “memory disorder*” OR “neurocognitive disorder*” OR neurodegenerat* OR (cogniti* N3 (declin* OR defect* OR disorder* OR impair* OR dysfunction* OR perform* OR function* OR health OR aging OR reserve OR resilien*))) AND (longitudinal* OR predict* OR prospective* OR “follow up” OR retrospective* OR wave OR time*point OR traject* OR “over*time” OR cohort* OR incidence OR “hazard ratio”) ) | 49,397 |
|  | **#9** | **#7 OR #8** | 54,993 |
| **Combined** | **#10** | **#3 AND #6 AND #9** | 542 |

### **PsycINFO**

| **Main Concepts** | **Search** | **Query** | **Results retrieved** |
| --- | --- | --- | --- |
| **Cardiometabolic Multimorbidity (CMM)** | **#1** | **Index terms:**  (DE "Cardiovascular Risk" OR DE "Cardiovascular Health" OR DE "Cardiovascular Disorders" OR DE "Comorbidity") AND (MM "Hypertension" OR MM "Diabetes Mellitus" OR MM "Lipid Metabolism Disorders" OR MM "Cerebrovascular Accidents" OR MM "Heart Disorders" OR MM "Angina Pectoris" OR MM "Coronary Heart Disease" OR MM "Coronary Thromboses" OR MM "Heart Arrhythmias" OR MM "Myocardial Infarctions") | 3,343 |
|  | **#2** | **Keywords (title or abstract):**  TI ( (cardiometabolic* OR cardiovascular* OR metabolic* OR multimorbid* OR comorbid* OR "multiple chronic conditions") AND (stroke* OR “heart disease*” OR “angina pectoris” OR “heart arrhythmia*” OR “cardiac arrhythmia*” OR “atrial fibrillation” OR “heart failure” OR “heart infarction” OR hyperlipid* OR dyslipid* OR diabet* OR hypertensi*) ) OR AB ( (cardiometabolic* OR cardiovascular* OR metabolic* OR multimorbid* OR comorbid* OR "multiple chronic conditions") AND (stroke* OR “heart disease*” OR “angina pectoris” OR “heart arrhythmia*” OR “cardiac arrhythmia*” OR “atrial fibrillation” OR “heart failure” OR “heart infarction” OR hyperlipid* OR dyslipid* OR diabet* OR hypertensi*) ) | 20,213 |
|  | **#3** | **#1 OR #2** | 21,890 |
| **Lifestyles** | **#4** | **Index terms:**  MM "Lifestyle" OR MM "Active Living" OR MM "Lifestyle Changes" OR MM "Health Behavior" OR MM "Exercise Dependence" OR MM "Health Risk Behavior" OR MM "Hygiene" OR MM "Preventive Health Behavior" OR MM "Self-Care" OR MM "Physical Activity" OR MM "Exercise" OR MM "Aerobic Exercise" OR MM "Weightlifting" OR MM "Yoga" OR MM "Eating Behavior" OR MM "Healthy Eating" OR MM "Diets" OR MM "Nutrition" OR MM "Alcohol Use" OR MM "Alcoholic Beverages" OR MM "Tobacco Smoking" OR MM "Sleep" | 200,817 |
|  | **#5** | **Keywords (title or abstract):**  TI (lifestyle* OR exercis* OR "physical* activ*" OR "physical* inactiv*" OR sport* OR sedentary OR diet* OR nutrition* OR "alcohol use" OR "tobacco use" OR sleep* OR leisure* OR recreation* OR “social connect*” OR “social engag*” OR "social network*" OR "social activit*" OR ((health OR eating OR drinking OR smoking) N2 (behav* OR habit*)) ) OR AB ( lifestyle* OR exercis* OR "physical* activ*" OR "physical* inactiv*" OR sport* OR sedentary OR diet* OR nutrition* OR "alcohol use" OR "tobacco use" OR sleep* OR leisure* OR recreation* OR “social connect*” OR “social engag*” OR "social network*" OR "social activit*" OR ((health OR eating OR drinking OR smoking) N2 (behav* OR habit*))) | 493,775 |
|  | **#6** | **#4 OR #5** | 548,938 |
| **Cognitive decline in longitudinal observational studies** | **#7** | **Index terms:**  (DE "Neurodegenerative Diseases" OR DE "Neurocognitive Disorders" OR DE "Alzheimer's Disease" OR DE "Dementia with Lewy Bodies" OR DE "Vascular Dementia" OR DE "Cognitive Impairment" OR DE "Mild Cognitive Impairment" OR DE "Dementia" OR DE "Senile Dementia" OR DE "Cognitive Ability" OR DE "Reading Ability" OR DE "Spatial Ability" OR DE "Verbal Ability" OR DE "Executive Function" OR DE "Memory Disorders" OR DE "Cognitive Aging" OR DE "Cognitive Reserve" OR DE "Resilience (Psychological)") AND (DE "Longitudinal Studies" OR DE "Prospective Studies" OR DE "Followup Studies" OR DE "Retrospective Studies" OR DE "Cohort Analysis") | 13,773 |
|  | **#8** | **Keywords (title or abstract):**  TI ( (dementia* OR alzheimer* OR “mild cognitive impairment*” OR “lewy body disease” OR “brain age” OR “memory loss” OR “memory disorder*” OR “neurocognitive disorder*” OR neurodegenerat* OR (cogniti* N3 (declin* OR defect* OR disorder* OR impair* OR dysfunction* OR perform* OR function* OR health OR aging OR reserve OR resilien*))) AND (longitudinal* OR predict* OR prospective* OR “follow up” OR retrospective* OR wave OR time*point OR traject* OR “over*time” OR cohort* OR incidence OR “hazard ratio”) ) OR AB ( (dementia* OR alzheimer* OR “mild cognitive impairment*” OR “lewy body disease” OR “brain age” OR “memory loss” OR “memory disorder*” OR “neurocognitive disorder*” OR neurodegenerat* OR (cogniti* N3 (declin* OR defect* OR disorder* OR impair* OR dysfunction* OR perform* OR function* OR health OR aging OR reserve OR resilien*))) AND (longitudinal* OR predict* OR prospective* OR “follow up” OR retrospective* OR wave OR time*point OR traject* OR “over*time” OR cohort* OR incidence OR “hazard ratio”) ) | 71,860 |
|  | **#9** | **#7 OR #8** | 77,247 |
| **Combined** | **#10** | **#3 AND #6 AND #9** | 392 |

### **Web of Science**

| **Main Concepts** | **Search** | **Query** | **Results retrieved** |
| --- | --- | --- | --- |
| **CMM** | **#1** | TS= ((cardiometabolic* OR cardiovascular* OR metabolic* OR multimorbid* OR comorbid* OR "multiple chronic conditions") AND (stroke* OR “heart disease*” OR “angina pectoris” OR “heart arrhythmia*” OR “cardiac arrhythmia*” OR “atrial fibrillation” OR “heart failure” OR “heart infarction” OR hyperlipid* OR dyslipid* OR diabet* OR hypertensi*)) | 2,768,656 |
| **Lifestyles** | **#2** | TS=(lifestyle* OR exercis* OR "physical* activ*" OR "physical* inactiv*" OR sport* OR sedentary OR diet* OR nutrition* OR "alcohol use" OR "tobacco use" OR sleep* OR leisure* OR recreation* OR “social connect*” OR “social engag*” OR "social network*" OR "social activit*" OR ((health OR eating OR drinking OR smoking) NEAR/2 (behav* OR habit*))) | 8,938,144 |
| **Cognitive decline in longitudinal observational studies** | **#3** | TS= ((dementia* OR alzheimer* OR “mild cognitive impairment*” OR “lewy body disease” OR “brain age” OR “memory loss” OR “memory disorder*” OR “neurocognitive disorder*” OR neurodegenerat* OR (cogniti* NEAR/3 (declin* OR defect* OR disorder* OR impair* OR dysfunction* OR perform* OR function* OR health OR aging OR reserve OR resilien*))) AND (longitudinal* OR predict* OR prospective* OR "follow up" OR retrospective* OR wave OR time*point OR traject* OR “over*time” OR cohort* OR incidence OR “hazard ratio”)) | 344,249 |
| **Combined** | **#4** | **#1 AND #2 AND #3** | 9,359 |

### **Scopus**

| **Main Concepts** | **Search** | **Query** | **Results retrieved** |
| --- | --- | --- | --- |
| **CMM** | **#1** | TITLE-ABS-KEY ( ( cardiometabolic* OR cardiovascular* OR metabolic* OR multimorbid* OR comorbid* OR "multiple chronic conditions" ) AND ( stroke* OR "heart disease*" OR "angina pectoris" OR "heart arrhythmia*" OR "cardiac arrhythmia*" OR "atrial fibrillation" OR "heart failure" OR "heart infarction" OR hyperlipid* OR dyslipid* OR diabet* OR hypertensi* ) ) | 989,960 |
| **Lifestyles** | **#2** | TITLE-ABS-KEY ( lifestyle* OR exercis* OR "physical* activ*" OR "physical* inactiv*" OR sport* OR sedentary OR diet* OR nutrition* OR "alcohol use" OR "tobacco use" OR sleep* OR leisure* OR recreation* OR "social connect*" OR "social engag*" OR "social network*" OR "social activit*" OR ( ( health OR eating OR drinking OR smoking ) near/2 ( behav* OR habit* ) ) ) | 4,525,226 |
| **Cognitive decline in longitudinal observational studies** | **#3** | TITLE-ABS-KEY ( ( dementia* OR alzheimer* OR "mild cognitive impairment*" OR "lewy body disease" OR "brain age" OR "memory loss" OR "memory disorder*" OR "neurocognitive disorder*" OR neurodegenerat* OR ( cogniti* AND near/3 ( declin* OR defect* OR disorder* OR impair* OR dysfunction* OR perform* OR function* OR health OR aging OR reserve OR resilien* ) ) ) AND ( longitudinal* OR predict* OR prospective* OR "follow up" OR retrospective* OR wave OR time*point OR traject* OR "over*time" OR cohort* OR incidence OR "hazard ratio" ) ) | 153,784 |
| **Combined** | **#4** | **#1 AND #2 AND #3** | 3,314 |
