# Supplementary material for: The role of lifestyle in mitigating cognitive decline in older adults with cardiometabolic multimorbidity (CMM): A protocol of systematic review and meta-analysis
Source: PLoS One. 2025 Dec 18;20(12):e0338896. doi: 10.1371/journal.pone.0338896 (PMC12714272; doi:10.1371/journal.pone.0338896)
Supplement: S3 File — (DOCX) [file pone.0338896.s003.docx]

**The role of lifestyle in mitigating cognitive decline in older adults with cardiometabolic multimorbidity (CMM): A protocol of systematic review and meta-analysis**

# Research question

Does a healthier lifestyle mitigate the detrimental effects of CMM on cognitive health?

# R code (with a simulated dataset)

**# 0. Load packages**

required_pkgs <- c("metafor", "dplyr", "ggplot2", "readr")

new_pkgs <- required_pkgs[!(required_pkgs %in% installed.packages()[, "Package"])]

if(length(new_pkgs)) install.packages(new_pkgs, dependencies = TRUE)

library(metafor)

library(dplyr)

library(ggplot2)

library(readr)

**# 1. Simulate dataset**

n_studies <- 18

dat <- data.frame(

study_id = paste0("Study_", 1:n_studies),

measure = sample(c("HR", "OR", "RR", "BETA"), n_studies, replace = TRUE,

prob = c(0.4, 0.25, 0.15, 0.2)),

effect = round(runif(n_studies, 0.65, 1.25), 3),

lower_CI = NA, upper_CI = NA,

sample_size = sample(seq(500, 5000, by = 200), n_studies, replace = TRUE),

followup_years = sample(2:12, n_studies, replace = TRUE),

lifestyle_type = sample(1:3, n_studies, replace = TRUE),

risk_of_bias = sample(c("low", "moderate", "high"), n_studies, replace = TRUE,

prob = c(0.4, 0.4, 0.2))

)

# Simulate CI widths depending on effect type

for (i in 1:n_studies) {

if (dat$measure[i] == "BETA") {

dat$effect[i] <- round(runif(1, -0.35, 0.10), 3)

se_b <- runif(1, 0.04, 0.12)

dat$lower_CI[i] <- dat$effect[i] - 1.96 * se_b

dat$upper_CI[i] <- dat$effect[i] + 1.96 * se_b

} else {

se_log <- runif(1, 0.05, 0.15)

dat$lower_CI[i] <- round(dat$effect[i] * exp(-1.96 * se_log), 3)

dat$upper_CI[i] <- round(dat$effect[i] * exp( 1.96 * se_log), 3)

}

}

print(dat)

**# 2. Convert effect measures to Hedges' g[1,2]**

# Treat HR/OR/RR as equivalent (log scale) given low incidence of dementia

# g = log(OR/HR/RR) * sqrt(3)/π

# g = β * π/√3

# var(g) derived from SE of log(effect) or β

convert_to_g <- function(effect, lower, upper, measure){

if (is.na(effect) | is.na(lower) | is.na(upper) | is.na(measure)) {

return(c(NA, NA))

}

measure <- toupper(trimws(measure))

if (measure %in% c("HR","OR","RR")) {

# log(effect) -> standardized mean difference (d)

logE <- log(effect)

se_log <- (log(upper) - log(lower)) / (2 * 1.96)

g <- logE * (sqrt(3) / pi)

var_g <- (se_log^2) * (3 / pi^2)

} else if (measure == "BETA") {

# standardized beta -> approximate d

g <- effect * (pi / sqrt(3))

se_beta <- (upper - lower) / (2 * 1.96)

se_g <- se_beta * (pi / sqrt(3))

var_g <- se_g^2

} else {

g <- NA; var_g <- NA

}

return(c(g, var_g))

}

conv <- t(mapply(convert_to_g, dat$effect, dat$lower_CI, dat$upper_CI, dat$measure, SIMPLIFY = TRUE))

dat$yi <- as.numeric(conv[,1])

dat$vi <- as.numeric(conv[,2])

# Keep only rows with valid yi/vi

dat <- dat %>% filter(!is.na(yi) & !is.na(vi))

if(nrow(dat) == 0) stop("No valid effect sizes after conversion. Check your input file.")

message("Converted effect sizes (first rows):")

print(dat %>% select(study_id, measure, effect, lower_CI, upper_CI, yi, vi) %>% head())

**# 3. Meta-analysis: Random-effects (REML) with HKSJ CIs**

# Use rma() with test="knha" for HKSJ-adjusted CIs

res <- rma(yi = yi, vi = vi, data = dat, method = "REML", test = "knha", slab = dat$study_id)

message("Meta-analysis (REML + HKSJ) summary:")

print(summary(res))

**# 4. Heterogeneity & prediction interval**

pred <- predict(res) # prediction interval on g scale

tau2 <- res$tau2

I2 <- res$I2

message(sprintf("tau^2 = %.4f ; I^2 = %.1f%%", tau2, I2))

message(sprintf("95%% prediction interval (g): %.3f to %.3f", pred$pi.lb, pred$pi.ub))

**# 5. Forest plot**

# Create a publication-ready forest plot (Hedges' g scale)

png(filename = "forest_meta_Hedges_g.png", width = 900, height = 1100, res = 150)

forest(res,

slab = dat$study_id,

xlab = "Hedges' g (negative = faster decline)",

mlab = "(REML + HKSJ pooled)",

cex = 0.9)

addpoly(res, row = -1, mlab = "(REML + HKSJ pooled)")

dev.off()

message("Forest plot saved to: forest_meta_Hedges_g.png")

**# 6. Meta-regression (example moderator: follow-up years)**

if("followup_years" %in% names(dat) && !all(is.na(dat$followup_years))){

message("Running meta-regression with followup_years as moderator...")

res_mreg <- rma(yi = yi, vi = vi, mods = ~ followup_years, data = dat,

method = "REML", test = "knha")

print(summary(res_mreg))

} else {

message("No followup_years moderator available or all NA; skipping meta-regression.")

}

**# 7. Sensitivity analyses**

**# 7.1 Leave-one-out**

message("Leave-one-out analysis:")

loo <- leave1out(res)

print(loo)

**# 7.2 HR-only sensitivity (if at least 3 HR studies)**

if("measure" %in% names(dat) && sum(dat$measure == "HR", na.rm = TRUE) >= 3){

dat_HRonly <- filter(dat, measure == "HR")

res_HRonly <- rma(yi = yi, vi = vi, data = dat_HRonly, method = "REML", test = "knha")

message("Sensitivity: HR-only pooled (REML + HKSJ):")

print(summary(res_HRonly))

} else {

message("Not enough HR-only studies to run HR-only sensitivity.")

}

**# 7.3 Exclude studies with follow-up < 5 years**

if("followup_years" %in% names(dat)){

dat_longfu <- filter(dat, followup_years >= 5)

message(sprintf("Studies with follow-up >=5 years: %d", nrow(dat_longfu)))

if(nrow(dat_longfu) >= 3){

res_longfu <- rma(yi = yi, vi = vi, data = dat_longfu, method = "REML", test = "knha")

message("Sensitivity: follow-up >=5 years pooled (REML + HKSJ):")

print(summary(res_longfu))

} else {

message("Not enough studies with follow-up >= 5 years.")

}

} else {

message("followup_years not available in dataset; skipping follow-up sensitivity.")

}

**# 7.4 Exclude high risk-of-bias studies**

if("risk_of_bias" %in% names(dat)){

dat_nohighrob <- filter(dat, risk_of_bias != "high")

message(sprintf("Studies after excluding high ROB: %d", nrow(dat_nohighrob)))

if(nrow(dat_nohighrob) >= 3){

res_nohigh <- rma(yi = yi, vi = vi, data = dat_nohighrob, method = "REML", test = "knha")

message("Sensitivity: excluding high risk-of-bias (REML + HKSJ):")

print(summary(res_nohigh))

} else {

message("Not enough studies remain after excluding high risk-of-bias.")

}

} else {

message("risk_of_bias column not available; skipping ROB sensitivity.")

}

**# 8. Back-transform pooled Hedges' g to HR scale**

# Relationship used: log(HR) ~ g * pi / sqrt(3)

g_pooled <- coef(res) # pooled g

g_CI <- c(res$ci.lb, res$ci.ub)

logHR_est <- g_pooled * (pi / sqrt(3))

logHR_CI <- g_CI * (pi / sqrt(3))

HR_est <- exp(logHR_est)

HR_CI <- exp(logHR_CI)

message("\nBack-transformed to HR scale (approx.):")

message(sprintf("Approx. pooled HR = %.3f (95%% CI: %.3f - %.3f)", HR_est, HR_CI[1], HR_CI[2]))

**# 9. Publication bias**

if (res$k >= 10) {

png(filename = "funnel_meta_Hedges_g.png", width = 700, height = 700, res = 130)

funnel(res, xlab = "Hedges' g", main = "Funnel plot")

dev.off()

message("Funnel plot saved to: funnel_meta_Hedges_g.png")

egger <- regtest(res, model = "rma")

message("Egger's test for small-study effects:")

print(egger)

tf <- trimfill(res)

png(filename = "funnel_trimfill_Hedges_g.png", width = 700, height = 700, res = 130)

funnel(tf, xlab = "Hedges' g", main = "Trim-and-fill funnel plot")

dev.off()

message("Trim-and-fill funnel plot saved to: funnel_trimfill_Hedges_g.png")

} else {

message("Fewer than 10 studies — funnel plot and Egger’s test not recommended.")

}

**# 10. Export summary**

results_summary <- data.frame(

k = res$k,

pooled_g = as.numeric(g_pooled),

CI_lower_g = as.numeric(res$ci.lb),

CI_upper_g = as.numeric(res$ci.ub),

tau2 = as.numeric(res$tau2),

I2 = as.numeric(res$I2),

pooled_HR_equiv = HR_est,

HR_CI_lower = HR_CI[1],

HR_CI_upper = HR_CI[2]

)

write.csv(results_summary, "meta_pooled_summary_HRscale.csv", row.names = FALSE)

message("Results summary exported to: meta_pooled_summary_HRscale.csv")

print(results_summary)

# END

message("Meta-analysis protocol run complete.")

# Reference

1. Chinn S. A simple method for converting an odds ratio to effect size for use in meta-analysis. Statist Med. 2000;19: 3127–3131. doi:10.1002/1097-0258(20001130)19:22%3C3127::AID-SIM784%3E3.0.CO;2-M

2. Lassale C, Batty GD, Baghdadli A, Jacka F, Sánchez-Villegas A, Kivimäki M, et al. Healthy dietary indices and risk of depressive outcomes: A systematic review and meta-analysis of observational studies. Mol Psychiatry. 2019;24: 965–986. doi:10.1038/s41380-018-0237-8
