# Supplementary material for: The role of lifestyle in mitigating cognitive decline in older adults with cardiometabolic multimorbidity (CMM): A protocol of systematic review and meta-analysis
Source: PLoS One. 2025 Dec 18;20(12):e0338896. doi: 10.1371/journal.pone.0338896 (PMC12714272; doi:10.1371/journal.pone.0338896)
Supplement: Attachment — Submitted filename: Response to reviewers-02102025.docx [file pone.0338896.s005.docx]

**Response to editors and reviewers**

Dear editors and reviewers,

We would like to sincerely thank you for your constructive comments and valuable feedback on our manuscript. Your suggestions have greatly helped us to improve the clarity, rigor, and compliance with the journal’s requirements. We have carefully revised the manuscript and addressed each point raised. Below, we provided a detailed, point-by-point response to the comments.

**Response to the editor**

Journal Requirements:
When submitting your revision, we need you to address these additional requirements.

1. Please ensure that your manuscript meets PLOS ONE's style requirements, including those for file naming. The PLOS ONE style templates can be found at

https://journals.plos.org/plosone/s/file? id=wjVg/PLOSOne_formatting_sample_main_body.pdf and https://journals.plos.org/plosone/s/file? id=ba62/PLOSOne_formatting_sample_title_authors_affiliations.pdf

We checked carefully and ensured that the manuscript meets PLOS ONE style requirements.

2. Thank you for stating in your Funding Statement:

[This work was supported by the Trinity College Dublin-China Scholarship Council PhD Scholarship (Grant Number 202406920021). The funders had no role in study design, data collection and analysis, decision to publish, or preparation of the manuscript.].

Please provide an amended statement that declares *all* the funding or sources of support (whether external or internal to your organization) received during this study, as detailed online in our guide for authors at <http://journals.plos.org/plosone/s/submit-now>.Please also include the statement “There was no additional external funding received for this study.” in your updated Funding Statement.

Please include your amended Funding Statement within your cover letter. We will change the online submission form on your behalf.

3. Thank you for stating the following in your manuscript:

[This work was supported by the Trinity College Dublin-China Scholarship Council PhD Scholarship (Grant Number 202406920021). The funders had no role in study design, data collection and analysis, decision to publish, or preparation of the manuscript.]

We note that you have provided funding information that is currently declared in your Funding Statement. However, funding information should not appear in the Acknowledgments section or other areas of your manuscript. We will only publish funding information present in the Funding Statement section of the online submission form.

Please remove any funding-related text from the manuscript and let us know how you would like to update your Funding Statement. Currently, your Funding Statement reads as follows:

[JS received the Trinity College Dublin-China Scholarship Council PhD Scholarship (Grant Number 202406920021). The funders had no role in study design, data collection and analysis, decision to publish, or preparation of the manuscript.]

Please include your amended statements within your cover letter; we will change the online submission form on your behalf.

We have removed the funding information from the manuscript and amended the Funding Statement as follows in the cover letter:

“JS received the Trinity College Dublin-China Scholarship Council PhD Scholarship (Grant Number 202406920021). The funders had no role in study design, data collection and analysis, decision to publish, or preparation of the manuscript. Proof of an independent peer review by the funder is not applicable in this case, as the funding was awarded as a general PhD scholarship. There was no additional external funding received for this study.”

We appreciate your assistance in updating the online submission form on our behalf.

4. When completing the data availability statement of the submission form, you indicated that you will make your data available on acceptance. We strongly recommend all authors decide on a data sharing plan before acceptance, as the process can be lengthy and hold up publication timelines. Please note that, though access restrictions are acceptable now, your entire data will need to be made freely accessible if your manuscript is accepted for publication. This policy applies to all data except where public deposition would breach compliance with the protocol approved by your research ethics board. If you are unable to adhere to our open data policy, please kindly revise your statement to explain your reasoning, and we will seek the editor's input on an exemption. Please be assured that, once you have provided your new statement, the assessment of your exemption will not hold up the peer review process.

Thank you for your guidance regarding the data availability statement. We agree with the journal’s open data policy and will ensure that all data is made freely accessible upon acceptance for publication.

5. Your ethics statement should only appear in the Methods section of your manuscript. If your ethics statement is written in any section besides the Methods, please move it to the Methods section and delete it from any other section. Please ensure that your ethics statement is included in your manuscript, as the ethics statement entered into the online submission form will not be published alongside your manuscript.

If the reviewer comments include a recommendation to cite specific previously published works, please review and evaluate these publications to determine whether they are relevant and should be cited. There is no requirement to cite these works unless the editor has indicated otherwise.

We have ensured that the ethics statement now only appears under the Method section “Ethics and dissemination” of the manuscript.

**Response to reviewers**

**Reviewer #1**

Song and colleagues are planning to perform a systematic review and meta-analysis, here outlining their planned work in an overall well-written and constructed protocol. A PROSPERO version of the protocol is available, and as of the 10th of July, it appears as if the work is at the stage of screening. Preferably, the authors should have waited for at least the initial reviewer feedback before commencing the formal search and screening, but it is, at the same time, fully understandable that they have now, and in practical terms, this is often the reality. The topic is clinically relevant, with a novel angle, and I applaud the authors for submitting a detailed protocol! In general, I see no major issues with this protocol or the methodological approaches outlined; however, there are bits here and there that can benefit from some polishing and revision. I've outlined those below. Please apologize for the many points, some of them are relatively trivial and have more the nature of questions.

Thank you for your detailed and constructive comments. We sincerely appreciate your recognition of the clinical relevance and novelty of our topic.

Regarding the timing of the formal search and screening, we acknowledge your point. However, given the typically long waiting time for initial review feedback, we deemed it more practical to initiate the formal search and screening in advance. These processes, however, were conducted under the guidance of a librarian, and the research team received appropriate training to ensure methodological rigor.

We are grateful for your careful review and for highlighting areas that could benefit from further refinement. We carefully addressed all comments and revised the manuscript accordingly.

**PROSPERO** (just three trivial questions)
- why did you switch the author order (between the PROSPERO protocol and this protocol)? is any of the authors a methodologist (in meta-research/evidence synthesis)/librarian, or has a person with such competence been consulted throughout the planning of this SR/MA? (e.g., for double-checking the search queries or the like)

Sorry for the confusion this caused. Yes, there is a librarian involved in our project. The initial PROSPERO entry did not strictly follow the intended author order, and we amended the order in the manuscript to ensure consistency.

- Is the "Current review stage" indeed up-to-date? Because, as I see, the first three steps have been marked as "started", but neither of the first two has been marked as "completed"...

Thanks for pointing this out. However, the “current review stage” in PROSPERO is not always updated in real time. We have now updated the PROSPERO entry accordingly to reflect the current progress.

**Title**
- Why is the focus/(choice of word) on *mitigating* life-style factors? Usually, from what I see, lifestyle factors investigated are rather *risk* factors...

We understand the potential confusion. While many studies indeed investigate lifestyles as risk factors, our review specifically focuses on their mitigating role, examining how they act as moderators or mediators that lessen the adverse effects of CMM on cognitive outcomes. We therefore chose the term *mitigating* to better reflect the scope and purpose of our review.

However, your comments prompted us to critically reconsider, and we now realise that the revised wording aligns better with the aim of this review. The change of title is consistent with our research question and previous search strategies.

Line 1-2:

Title changed:

“*The role of lifestyle in mitigating cognitive decline in older adults with cardiometabolic multimorbidity (CMM): A protocol of systematic review and meta-analysis”*

**Abstract**
- From the Background, it's not clear why a SR/MA is needed. Is it because the results from primary studies are conflicting? Is it that it's unclear which *specific* cognitive outcomes the associations are actually robust/consistent in? Something else?

Thank you for this comment. We have revised the background to clarify the rationale for conducting this SR/MA.

Line 22-27:

“Cardiometabolic multimorbidity (CMM), the coexistence of two or more cardiometabolic diseases (CMDs), is increasingly recognized as a key risk factor for cognitive decline and dementia in older adults. Healthy lifestyle behaviors may mitigate these effects, but this remains poorly understood. Given the rising prevalence of CMM worldwide and the urgent need for effective dementia prevention strategies, a systematic review and meta-analysis are warranted to investigate whether and to what extent a healthier lifestyle can mitigate this risk.”

- In Methods, you write "[...] in older adults with CMM"... but what's the control group then? Please clarify/specify this part

Thank you for your comment. We phrased it this way to align with the main research terms. Our focus is on the primary research question, which compares individuals with less healthy lifestyles to those with healthier lifestyles, within the context of CMM.

No changes made.

- In Methods, you write about two independent reviewers doing screening, but what about data extraction and quality assessment? Those should be performed in pairs as well

Yes, data extraction and quality assessment will also be conducted by two reviewers independently. We explained this in the Method section; however, it was not included in the abstract due to word limits.

Line 32-33:

“Retrieved records will be managed in Covidence, with two independent reviewers conducting all review processes.”

**Introduction**
- Please clarify the difference between cognitive decline and cognitive impairment

Thank you for your comments.

Cognitive decline refers to a gradual deterioration in cognitive abilities over time, which may occur in normal aging or precede more serious deficits. Cognitive impairment denotes a measurable deficit at a single point in time, ranging from mild cognitive impairment (MCI) to dementia, and can constitute a categorial clinical diagnosis. Cognitive impairment can be defined as reaching a prespecified criterion of cognitive decline.

We have revised the text and added references to clarify this point.

Line 50-51:

“People with CMDs face an increased risk of developing cognitive decline, cognitive impairment with no dementia (CIND) [2], and ultimately dementia [3–5].”

- [2]. Ailshire JA, Farina MP, Jackson H, Walsemann KM. The role of educational attainment and quality in U.S. regional variation in prevalence of dementia and CIND. PLOS ONE. 2025;20: e0332410. doi:10.1371/journal.pone.0332410
- [5]. Vishwanath S, Qaderi V, Steves CJ, Reid CM, Hopper I, Ryan J. Cognitive decline and risk of dementia in individuals with heart failure: A systematic review and meta-analysis. J Card Fail. 2022;28: 1337–1348. doi:10.1016/j.cardfail.2021.12.014

- I'd simplify line 61 to "two-fold"/"twice"

Thank you for your advice. We aimed to present the exact evidence by reporting the numerical hazard ratios. However, we have adopted your suggestion and revised the expression to “two-fold” for simplicity.

Line 57-58:

“…have nearly a two-fold risk of developing dementia compared to those without multimorbidity [11].”

- The sentence on lines 66-68 feels unfinished..

You are right. We have revised and restructured the sentence.

Line 68-72:

“Many studies have investigated cognitive health using composite cardiovascular health scores, including indicators of both CMDs and lifestyle factors such as exercise, diet, and sleep [15,16]. While composite scores are useful for predicting cognitive outcomes in the general population, they may offer limited insight for tailoring interventions to individuals with CMM.”

- For the part on lines 88-93, please add country and mean age at baseline where it's missing

Thank you for your advice. We have revised the text and added the missing information regarding country and mean age to ensure clearer comparisons.

Line 60-64:

“Similarly, a 12-year follow-up study of Swedish older adults aged 60 and older found a slightly lower HR of 1.86 (95% CI 1.17 to 2.97) [17]. While data from the Health and Retirement Study, with a mean follow-up of 14.6 years, showed a higher HR of 3.27 (95% CI 2.06 to 5.21) in American participants with a mean age of 59.4 years [18].”

- On line 97, is it meant "[...] between CMM and cognitive outcomes [...]"?

Yes. We have changed accordingly.

Line 94:

“…CMM on cognitive health”

- Wouldn't the sentence on lines 100-102 be better suited in the very beginning of that paragraph?

You are correct. We have made the changes accordingly, moving the sentence “To date, no systematic review has synthesized the data regarding the effect of CMM on cognitive outcomes in older adults or the potential mitigating role of lifestyle factors in this relationship” to the beginning of the paragraph. (Line 89-90)

- Technically speaking (regarding lines 103-104), Alzheimer's disease is also a type of dementia.. So maybe just "dementia"? Why limit yourselves in that regard?

You are right. We have deleted “Alzheimer’s disease”.

Line 95:

“…including cognitive decline, CIND, and dementia”

- Again, on line 104, "[...] among older individuals with CMM [...]"... then who is the control group?

We hope our previous response to the same question provides clarification on this point.

- Finally, on line 104, "[...] and 2) Identify [...]"... I think what you mean is to investigate? Also, again, I understand completely the interest in healthy lifestyle factors to adopt, but what about modifiable *risk* factors that can be avoided? it sounds mostly comprehensive when reading your text, but at times quite limiting (what you mean)...

Thank you for this comment. We believe the confusion arose from the use of the word “identify”. You are correct that “investigate” is a more accurate term than “identify” in this context.

Line 93: substitute “identify” with “investigate”

**Methods**
- Which translation tools will be employed, and why?

Google Translate will be used when necessary, and we have added the relevant information with references to clarify this in the text.

Line 116-118:

“Studies published in all languages will be included to ensure methodological rigor, with Google Translate employed when necessary as a viable and accurate tool for translating in systematic reviews [23,24].”

- [23]. Davidson SL, Lee J, Emmence L, Bickerstaff E, Rayers G, Davidson E, et al. Systematic review and meta-analysis of the prevalence of frailty and pre-frailty amongst older hospital inpatients in low- and middle-income countries. Age Ageing. 2025;54: afae279. doi:10.1093/ageing/afae279
- [24]. Jackson JL, Kuriyama A, Anton A, Choi A, Fournier J-P, Geier A-K, et al. The accuracy of Google Translate for abstracting data from non–English-language trials for systematic reviews. Ann Intern Med. 2019;171: 677–679. doi:10.7326/M19-0891

- How will you deal with Google Scholar, given that it may easily return enormous amounts of records? Usually, SR/MA take the first 200-300 records or so; how is your plan, and why?

Thank you for this comment. For Google Scholar, to manage the potentially large number of results, we will screen sequentially and stop once it becomes evident that no additional relevant articles are appearing, which is consistent with common practice in previous studies.

Line 114-116:

“Grey literature, including unpublished studies, will also be searched using sources such as Google Scholar, ProQuest, and medRxiv.”

- Including gray literature is very good! But I wonder, is OpenGrey even searchable/indexed? It was more or less de facto defunct last time I checked... How about ProQuest Dissertations and Theses, OAIster, etc...

You are right, and we encountered the same situation. Changes have been made based on the current progress and practice, and we have found that Google Scholar, ProQuest, and medRxiv are more practical.

Line 114-116:

“Grey literature, including unpublished studies, will also be searched using sources such as Google Scholar, ProQuest, and medRxiv.”

- I'd strongly recommend searching PubMed instead of Medline, as the former contains ALL of Medline-indexed records PLUS some preprints and other material that could potentially be relevant. Although Medline admittedly offers richer search syntax (e.g., NEAR/x), I'd argue that comprehensiveness outweighs in this case
- Any plans about ensuring non-Western inclusion? E.g., I see you plan to use Web of Science, which is great. Please consider also searching KCI and SciELO through WoS (as I recall, it doesn't search those by default, just the "core collection"). Also, please consider WHO Global Index Medicus, CABI Direct...

Thank you for your thoughtful recommendations for databases. We agree that PubMed offers broader coverage than Medline, including preprints and other records. However, after consulting with our experienced librarian, we selected the six databases to balance comprehensiveness and practicality, while separating common databases from grey literature sources for efficiency. We appreciate your suggestions on additional resources, and we will carefully consider these to enhance non-Western inclusion and minimize regional bias.

No changes made.

- I'm missing a search query for Google Scholar/OpenGrey

We will use the keywords “cardiometabolic multimorbidity AND cognition AND lifestyle.” Detailed search strategies were not included in the text due to word limits.

No changes made.

- Why the cut-off of ≥50 years (at baseline, I assume)?

Thank you for your question. We realized the explanation for the age cut-off was missing and have now added justifications, supported by relevant references.

Line 128-134:

“Population: Older adults aged 50 years or above (at baseline) with CMM, defined as the coexistence of ≥ 2 CMDs [26]. We set the age cut-off because adults aged 50 and over experience a high prevalence of multimorbidity, with over 80% affected [27], making this a critical window to examine the impact of CMD accumulation on cognitive health. Furthermore, as this population may not yet exhibit clinically significant cognitive impairment [28], studying the mitigating role of lifestyle factors is particularly relevant for informing future preventive strategies.”

- [27].	Nguyen H, Chua K-C, Dregan A, Vitoratou S, Bayes-Marin I, Olaya B, et al. Factors associated with multimorbidity patterns in older adults in England: Findings from the English longitudinal study of aging (ELSA). J Aging Health. 2020;32: 1120–1132. doi:10.1177/0898264319891026
- [28].	Bai W, Chen P, Cai H, Zhang Q, Su Z, Cheung T, et al. Worldwide prevalence of mild cognitive impairment among community dwellers aged 50 years and older: a meta-analysis and systematic review of epidemiology studies. Age Ageing. 2022;51(8):afac173. doi:10.1093/ageing/afac173

- Hmm, reading the Comparators section, it remains a bit unclear to me, what will *actually* be investigated.. because based on lines 147-149, you are not searching for studies with control groups being without (as many) cardiometabolic diseases... so are you indeed *only* interested in populations where ≥2 CMDs are present?

Thank you for your comments. We have simplified the research question and clarified the PECO framework to avoid potential confusion.

Line 139:

“Comparator: Individuals with less healthy lifestyles within the context of CMM.”

- Are you sure that case-control studies qualify as longitudinal? Judging by the ROBINS-E paper, it's also not exactly geared for it ("Future work will produce variants of the tool for other epidemiologic study designs (e.g., case-control studies)."; https://pmc.ncbi.nlm.nih.gov/articles/PMC11098530). To strengthen the rigor and clinical utility of this SR/MA, I'd refrain from including case-control studies and keep it at prospective cohort studies, historical cohort data, case-cohort studies, nested case-control studies (i.e., within a cohort), etc..

Thank you for your suggestion. What we intended to include were nested case-control studies conducted within a cohort. To avoid confusion and improve accuracy, we have revised the text to refer more clearly to cohort studies.

Line 145-146:

“Study design: Only studies using longitudinal observational design, such as prospective cohort studies, retrospective cohort studies, and nested case-control studies, will be included in this review.”

- Please reconsider excluding letters to the editor. At least from my experience, the trend in some high-impact journals recently is to downgrade many good-quality articles to letters. Some of the authors then opt for putting most of the now "extra" material/data to suppl. material and online repositories (thus, the same data and rigor/detail is actually there, as in a "full-length" article)...

Thank you for your advice, which we find reasonable. While letters, editorials, and comments are often considered opinion-based and may not contain formal data analyses, this is not always the case, just like you mentioned. If such articles contain relevant data or information, they will be considered in our review. However, we expect this to occur rarely in the context of our study.

Line 149-150:

“Additionally, studies that are not published as full reports, such as study protocols and editorials, will not be considered.”

- Will you employ any definition of minimum follow-up length? Would it make sense for some outcomes, given how slowly they develop? Or is this something that you'll simply just comment on, if you see fit?

Thank you for this insightful question. We will not set a minimum follow-up duration, given the anticipated limited number of eligible studies. However, we plan to conduct subgroup analyses or provide a discussion on follow-up duration depending on the results, as you suggested. These plans have been detailed in the “Data synthesis and statistical analysis” section.

No changes made.

- I'd place (and also *do*) Data Extraction prior to Assessment of methodological quality

Thank you for your advice. Change has been made accordingly.

- I assume you might want to revise the estimated deadline on line 197?

You are correct again.

Line 189:

“The data extraction is expected to be completed by October 2025.”

- I agree that HR ≈ RR in case the outcome is rare (<15% or so), but I would say it's not quite the case otherwise (please see Table 2 in <https://doi.org/10.7326/M16-2607>)

Thank you for your comment and for sharing the reading.

We read your sharing and additional research. We know that the E-value is applied to evaluate if the estimate is potentially biased by unmeasured confounding. When the outcome is relatively rare (< 15%) by the end of follow-up, OR and HR can be treated as RR when calculating the E value.

Reference:

- VanderWeele TJ, Ding P. Sensitivity Analysis in Observational Research: Introducing the E-Value. Ann Intern Med. 2017;167: 268–274. doi:[10.7326/M16-2607](https://doi.org/10.7326/M16-2607)
- Gaster T, Eggertsen CM, Støvring H, Ehrenstein V, Petersen I. Quantifying the impact of unmeasured confounding in observational studies with the E value. BMJ Med. 2023;2: e000366. doi:10.1136/bmjmed-2022-000366

However, for the main synthesis, the RRs and ORs reported from the original studies will be treated as equivalent to HRs due to the relatively low incidence of dementia. No conflicts were observed, and we have added references to support this approach.

Line 190-193:

“Relative risks (RRs) and odds ratios (ORs) reported in the original studies will be treated as equivalent to HRs, given the relatively low incidence of dementia [31].”

- [31]. Gao Y, Zhang Z, Song J, Gan T, Lin Y, Hu M, et al. Combined healthy lifestyle behaviours and incident dementia: A systematic review and dose–response meta-analysis of cohort studies. International Journal of Nursing Studies. 2024;156: 104781. doi:[10.1016/j.ijnurstu.2024.104781](https://doi.org/10.1016/j.ijnurstu.2024.104781)

- Please clarify (or rather, specify) the meta-analysis models you want to employ. I assume random-effects, although you specifically mention "mixed" modelling multiple times; what's the rationale, and what setup are you expecting? Which method of assessment of between-study variance, etc? Also, please consider looking into tau(2), as it's more robust than I2 for small sample sizes, if you fear such scenarios in particular
- Why meta-regression for RQ2? Do you really expect such data?

Thank you for your comments and suggestions. The extended mixed-effects framework represents a novel approach that accommodates complex random-effects structures. In response, to avoid potential vagueness, we have decided to remove this expression and instead provide additional details on the specific models we plan to use, as well as clarify the methods for assessing heterogeneity.

Line 192-201:

“Analyses will be performed on the log scale and pooled using random-effects models with restricted maximum likelihood (REML) [32]. To obtain more reliable uncertainty estimates under conditions of limited studies and substantial heterogeneity, we will apply the Hartung-Knapp-Sidik-Jonkman (HKSJ) method, which adjusts the standard error of the pooled effect and uses a t-distribution [33]. The primary research question will be addressed using meta-regression, modelling lifestyle indicators as study-level moderators of the association between CMM and cognitive outcomes. Where possible, dose-response meta-analysis will model continuous lifestyle measures.

Heterogeneity will be reported with HKSJ-adjusted CIs, alongside the I^2^ statistic and τ^2^ estimates. In addition to the pooled estimates, we will report 95% prediction intervals to reflect the expected range of effects in future studies, offering greater practical interpretability [34].

- Subgroup/sensitivity analyses seem fine, but why no sensitivity analysis excluding low-quality studies (for example)?

Thank you for your question. We do not plan to exclude low-quality studies from the main analysis due to the anticipated limited number of eligible studies and to maintain comprehensiveness. However, we should and will address study quality in sensitivity analysis to examine whether the results are robust when low-quality studies are excluded. Thus, we have added it to the 4^th^ sensitivity analysis.

Line 210:

“Fourth, we will exclude studies with a high risk of bias to examine the robustness of the pooled results.”

- Please consider making your R code publicly available, together with collected data (for meta-analysis); a great example/inspiration can be found here: <https://journals.plos.org/plosmedicine/article?id=10.1371/journal.pmed.1003731>

Thank you for your suggestion and for sharing this example. We agree that making R code and data publicly available will enhance the transparency and methodological rigor, and we consider it for our review.

**Discussion**
- Is there a reference or a further specified rationale for what is said about IPD MA on lines 256-258? My personal point of view is that the heterogeneity seen in the clinical context is so great that MAs in and of themselves are by default quite "rough" and always need to be examined cautiously, and I haven't seen proof of IPD MA making things better per se (in this context)

Thank you for sharing your point of view. We acknowledged restricted access to individual participant data (IPD) as a limitation to our review because IPD would allow more flexible modelling of covariates and subgroup analyses. We wish to clarify that this comment was not intended to imply that IPD meta-analyses would resolve heterogeneity, but rather to highlight the additional analytical opportunities that access to IPD can provide.

Line 236-238:

“Secondly, most studies report aggregate data, and access to individual participant data (IPD) is often restricted, limiting the ability to perform IPD meta-analyses for more flexible modelling of covariates and subgroup analyses [43].”

**Reviewer #2**

This is a well-structured manuscript from abstract to reference. But, the author did not include any tables or diagrams to show what was done to back up the analysis. No evidence of the research carried out was involved in this manuscript.

We appreciate the reviewer’s positive comments regarding the structure of the manuscript. At this stage, the PRISMA flow diagram is not yet available. However, we have attached the detailed search strategy and PRISMA-protocol checklist to provide transparency and support the planned analysis.
