# Supplementary material for: The role of lifestyle in mitigating cognitive decline in older adults with cardiometabolic multimorbidity (CMM): A protocol of systematic review and meta-analysis
Source: PLoS One. 2025 Dec 18;20(12):e0338896. doi: 10.1371/journal.pone.0338896 (PMC12714272; doi:10.1371/journal.pone.0338896)
Supplement: Attachment — Submitted filename: Response to reviewers-04112025.docx [file pone.0338896.s006.docx]

**Response to reviewers**

**Thank you very much for your comments. Please see our responses highlighted in blue.**

Additional Editor Comments:

Please further revise the manuscript to address all comments raised by the reviewer.

[Note: HTML markup is below. Please do not edit.]

Reviewers' comments:

Reviewer's Responses to Questions

**Comments to the Author**

1. Does the manuscript provide a valid rationale for the proposed study, with clearly identified and justified research questions?

The research question outlined is expected to address a valid academic problem or topic and contribute to the base of knowledge in the field.

Reviewer #1: Yes

Reviewer #2: Yes

2. Is the protocol technically sound and planned in a manner that will lead to a meaningful outcome and allow testing the stated hypotheses?

The manuscript should describe the methods in sufficient detail to prevent undisclosed flexibility in the experimental procedure or analysis pipeline, including sufficient outcome-neutral conditions (e.g. necessary controls, absence of floor or ceiling effects) to test the proposed hypotheses and a statistical power analysis where applicable. As there may be aspects of the methodology and analysis which can only be refined once the work is undertaken, authors should outline potential assumptions and explicitly describe what aspects of the proposed analyses, if any, are exploratory.

Reviewer #1: Yes

Reviewer #2: Yes

3. Is the methodology feasible and described in sufficient detail to allow the work to be replicable?

Descriptions of methods and materials in the protocol should be reported in sufficient detail for another researcher to reproduce all experiments and analyses. The protocol should describe the appropriate controls, sample size calculations, and replication needed to ensure that the data are robust and reproducible.

Reviewer #1: Yes

Reviewer #2: Yes

4. Have the authors described where all data underlying the findings will be made available when the study is complete?

The [PLOS Data policy](https://track.editorialmanager.com/CL0/https:%2F%2Fjournals.plos.org%2Fplosone%2Fs%2Fmaterials-and-software-sharing/1/010f0199ebd9647c-e3487895-bad9-424e-9637-5372db81b5d7-000000/Td3mpaIcfk_mHuQEx3VtltEM4bhOrI1vTfoDekmPVgo=233) requires authors to make all data underlying the findings described in their manuscript fully available without restriction, with rare exception, at the time of publication. The data should be provided as part of the manuscript or its supporting information, or deposited to a public repository. For example, in addition to summary statistics, the data points behind means, medians and variance measures should be available. If there are restrictions on publicly sharing data—e.g. participant privacy or use of data from a third party—those must be specified.

Reviewer #1: Yes

Reviewer #2: Yes

5. Is the manuscript presented in an intelligible fashion and written in standard English?

PLOS ONE does not copyedit accepted manuscripts, so the language in submitted articles must be clear, correct, and unambiguous. Any typographical or grammatical errors should be corrected at revision, so please note any specific errors here.

Reviewer #1: Yes

Reviewer #2: Yes

6. Review Comments to the Author

Please use the space provided to explain your answers to the questions above and, if applicable, provide comments about issues authors must address before this protocol can be accepted for publication. You may also include additional comments for the author, including concerns about research or publication ethics.

You may also provide optional suggestions and comments to authors that they might find helpful in planning their study.

(Please upload your review as an attachment if it exceeds 20,000 characters)

Reviewer #1: The authors have provided thorough and professional responses and appropriate revisions. I am happy.

Thank you for your positive feedback and for acknowledging our efforts. We sincerely appreciate your advice and guidance.

Reviewer #2: Thank you for your response. But, you only attached a checklist for the analysis and not the analysis itself, which do not suffice in my opinion, I understand your point about availability at the moment. You also need to know that, the analysis is important for your research paper.

Thank you for your comment.

We have carefully considered your previous feedback: “This is a well-structured manuscript from abstract to reference. But the author did not include any tables or diagrams to show what was done to back up the analysis. No evidence of the research carried out was involved in this manuscript.”

Given that the inclusion and exclusion criteria were clearly defined in the “Eligibility Criteria” section based on Population, Exposure, Comparator, and Outcomes (PECOS), we have additionally provided a partially completed PRISMA diagram (Fig. 1) alongside the R code to enhance clarity of the analyses. The R code has been validated using a simulation dataset to ensure reproducibility and is also accessible on GitHub (https://github.com/songj4-byte/HL_CMM_Cog_SRMA2025). Writing the R code helped clarify the analysis steps and some details. We also removed certain content to improve readability and understanding.

**Changes made:**

- Line 161: Added “(Fig. 1)”
- Line 196: Removed the sentence: “The primary research question will be addressed using meta-regression, modelling lifestyle indicators as study-level moderators of the association between CMM and cognitive outcomes.”

7. PLOS authors have the option to publish the peer review history of their article ([what does this mean?](https://track.editorialmanager.com/CL0/https:%2F%2Fjournals.plos.org%2Fplosone%2Fs%2Feditorial-and-peer-review-process%23loc-peer-review-history/1/010f0199ebd9647c-e3487895-bad9-424e-9637-5372db81b5d7-000000/GLywJtK23v1iUjsZ3H9JWXeI06za9KUEOGjHXPupDoA=233)). If published, this will include your full peer review and any attached files.

If you choose “no”, your identity will remain anonymous but your review may still be made public.

**Do you want your identity to be public for this peer review?** For information about this choice, including consent withdrawal, please see our [Privacy Policy](https://track.editorialmanager.com/CL0/https:%2F%2Fwww.plos.org%2Fprivacy-policy/1/010f0199ebd9647c-e3487895-bad9-424e-9637-5372db81b5d7-000000/0S3X3_bzXZwtK_tjEVE36CBqMpkvH34OKUulzhwVyrk=233).

Reviewer #1: **Yes:** Daniil Lisik

Reviewer #2: No
